# Artificial Intelligence-Driven Tea Quality Evaluation: A State-of-the-Art Review

**DOI:** 10.3390/foods15213981

**Published:** 2026-11-06

**Authors:** Ying Zhang, Jie Wang, Hongyu Luo, Rui Chang, Juan Yang, Riqi Duan, Yingfu Zhong

**Affiliations:** Chongqing Academy of Agricultural Sciences, Chongqing 401329, China; bellazy@126.com (Y.Z.); wjzhaiyang@163.com (J.W.); roye2008324@126.com (H.L.); cr94810@126.com (R.C.); yj20041539@163.com (J.Y.); duanruiqi@cqaas.cn (R.D.)

**Keywords:** artificial intelligence, deep learning, tea quality assessment, computer vision

## Abstract

Traditional tea quality evaluation relies largely on subjective human sensory assessment and destructive chemical laboratory assays, suffering from poor objectivity, low throughput and limited scalability. In the past five years, artificial intelligence-driven deep learning has enabled rapid, non-destructive, data-oriented quality inspection for various teas. This review summarizes representative advances covering computer vision, electronic nose, near-infrared spectroscopy, hyperspectral imaging, electronic tongue and multi-sensor multimodal fusion. CNNs, YOLO-series detectors, Transformer and CLIP achieve 85–99% classification accuracy in tea grading, aroma analysis, fermentation monitoring and geographical tracing, alongside accurate quantitative prediction of individual chemical constituents (e.g., R^2^ = 0.92–0.97 for four catechins in black tea). In most reviewed studies, multimodal frameworks achieved higher performance than single-sensor systems by exploiting complementary sensing information. However, industrial deployment is hindered by small non-standard datasets, low model interpretability, sensor drift, poor cross-domain transferability and high costs. Future directions include standardized benchmark datasets, explainable multimodal models, lightweight networks and compatible multi-sensor hardware platforms.

## 1. Introduction

Tea ranks among the most widely consumed beverages on a global scale, and its market economy attaches great commercial importance to product quality and authenticity verification [1,2,3,4,5]. Conventional tea quality assessment relies on two core methodological branches, namely human sensory evaluation and instrumental chemical quantification. Sensory assessment relies on trained expert panelists to evaluate five key quality dimensions under standardized protocols, covering dry leaf appearance, tea liquor color, fragrance, flavor taste, and infused leaf characteristics. Although this workflow reproduces the holistic sensory experience of tea consumption, it carries intrinsic subjectivity, is vulnerable to evaluator fatigue, and faces standardization barriers across different laboratories and seasonal conditions [6]. Analytical instruments including HPLC and GC-MS generate quantitative chemical profiles, yet these approaches are labor-intensive, costly and sample-destructive, making them unsuitable for real-time or on-line production-line quality supervision [6,7,8,9,10].

Global tea production exceeded 6.5 million tons in 2023, with China accounting for nearly 45% of the world output, making tea quality control a matter of significant economic and public health relevance. The international tea trade increasingly demands rapid, reliable, and non-destructive quality verification protocols that can operate at industrial throughput speeds. Consequently, the development of automated, intelligent tea quality evaluation systems has attracted substantial research attention from both academia and industry over the past decade. Artificial intelligence, particularly deep learning, has been successfully deployed across various food quality assessment domains including fruit grading, coffee quality evaluation, grain classification, and fresh-produce and meat quality monitoring, as well as flavor prediction in beer [10,11,12,13,14,15,16], demonstrating its potential for tea quality characterization.

Rapid advances in artificial intelligence, particularly deep learning algorithms, open new research avenues for tea quality characterization. Deep-learning-enabled models learn complex non-linear mapping relationships from multi-source sensor signals, bridging the gap between objective instrumental measurements and subjective sensory quality attributes. Deep-learning-based tea quality monitoring applications can be categorized into three sequential stages, spanning cultivation, manufacturing processing, and finished product inspection [17,18,19]. The integration of multimodal sensing hardware with deep learning facilitates a technical transition from subjective sensory judging towards rapid, non-destructive, and data-driven quality evaluation workflows [20,21].

The fundamental paradigm shift brought about by deep learning lies in its ability to automatically extract discriminative features from raw sensor data, eliminating the need for manual feature engineering that traditionally required domain expertise. Convolutional neural networks (CNNs) excel at capturing hierarchical spatial patterns from image data, making them particularly suited for visual tea appearance grading. Recurrent architectures and Transformers process sequential sensor responses from electronic noses and tongues, capturing temporal dynamics of aroma and taste release. Graph neural networks (GNNs) model relationships among multiple quality attributes, enabling holistic quality profiling. The contrastive language-image pre-training (CLIP) paradigm enables cross-modal alignment between visual appearance and semantic quality descriptions, pushing the boundary of expert-level tea grading [22,23,24]. A comparison of traditional and artificial intelligence-based tea quality evaluation methods is presented in Table 1.

**Figure 1:**
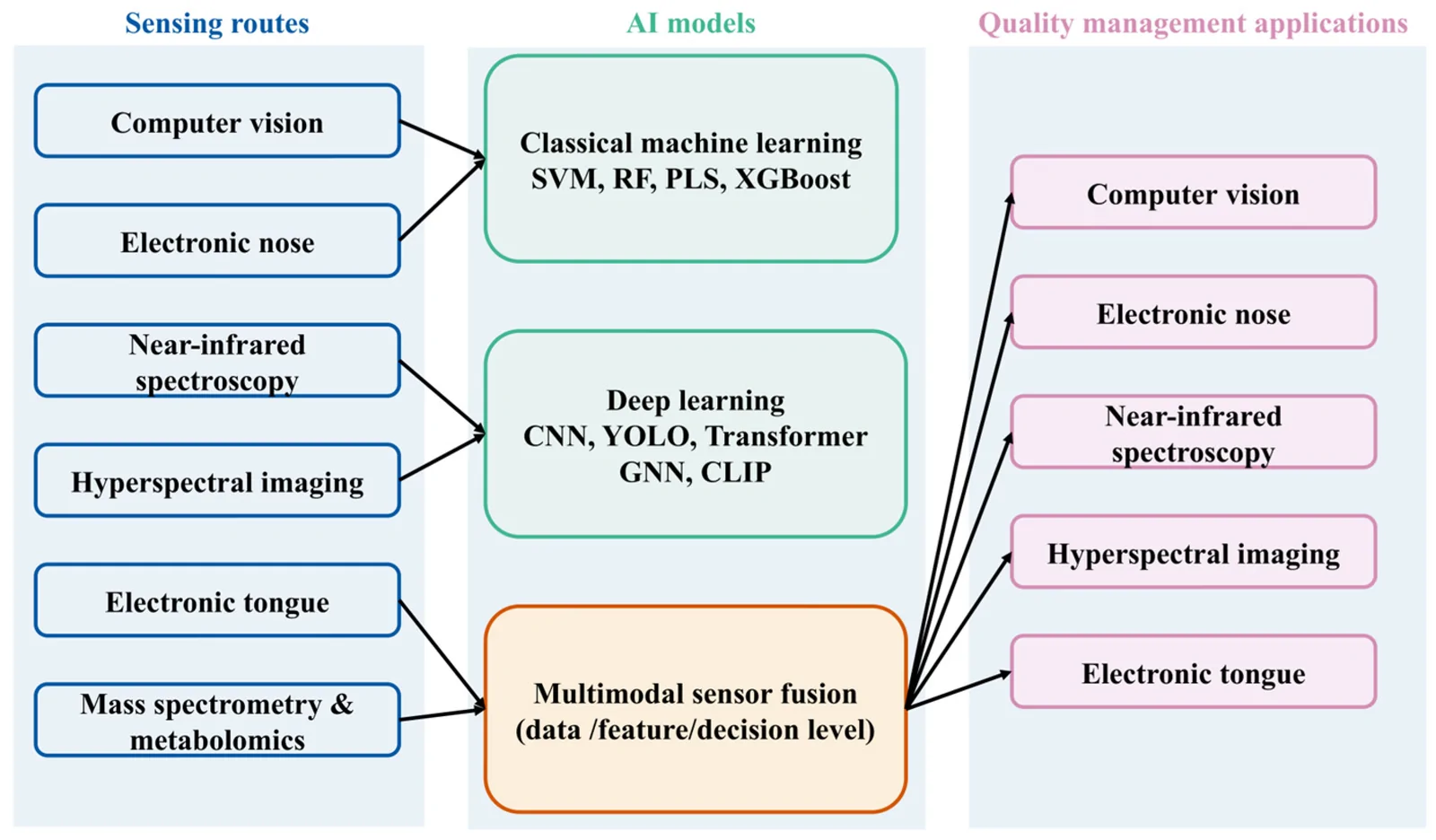
Overall framework of AI-assisted tea quality evaluation covered in this review. The six sensing routes comprise five single-modality routes (computer vision, electronic nose, near-infrared spectroscopy, hyperspectral imaging and electronic tongue) and multimodal fusion, which integrates combinations of these signals; the sensing routes provide complementary signals that feed classical machine-learning and deep-learning models to support diverse quality-management applications.

The present review consolidates peer-reviewed publications released between 2021 and 2026 retrieved from Web of Science, PubMed, IEEE Xplore, MDPI and CNKI databases, as shown in Figure 1. The literature search was first conducted in May 2026 using keyword combinations of tea quality and AI terms (e.g., ‘tea’ AND (‘grading’ OR ‘quality’ OR ‘origin’ OR ‘fermentation’) AND (‘deep learning’ OR ‘machine learning’ OR ‘computer vision’ OR ‘electronic nose’ OR ‘electronic tongue’ OR ‘hyperspectral’ OR ‘near-infrared’ OR ‘multimodal’)) across the five databases above, and was updated in August and September 2026 to capture articles published up to the date of resubmission (e.g., refs. [25,26]). Records were screened by title and abstract; original studies reporting quantitative tea quality assessment with AI methods were retained, whereas reviews were used only for contextual framing. This selection process yielded the 89 references finally cited in the revised manuscript. The primary coverage window is 2021–2026; a small number of seminal earlier studies (e.g., Refs. [27,28,29,30,31,32]) are retained because they provide foundational datasets and protocols that remain in active use in the primary literature. The present work is a narrative rather than a systematic review, and the selection process described above is intended to make it transparent how representative studies were identified rather than to claim exhaustive coverage. One deviation from the peer-reviewed criterion should be noted: Ref. [33], a doctoral thesis on electronic-tongue-based tea quality assessment, was retained because it provides foundational sensor-array and feature-extraction methodology that is widely relied upon in the peer-reviewed electronic-tongue literature; it is the only non-peer-reviewed source retained and is flagged. By categorizing the existing literature into six distinct technical routes, this work identifies mainstream research trends, major technical achievements and unresolved research gaps, aiming to deliver comprehensive references for follow-up researchers in this domain. In addition to surveying the six primary sensing modalities, this review provides a systematic analysis of deep learning architectures, data preprocessing pipelines, and model interpretability methods, accompanied by comprehensive comparison tables that facilitate cross-study benchmarking. Compared with recent AI-based tea quality reviews, which concentrate on either deep learning methods [17], individual sensing modalities [26], or multimodal sensor fusion [6], the present review advances the field by systematically benchmarking the six sensing routes (five single-modality routes and multimodal fusion) against a unified evaluation framework, explicitly analyzing data-preprocessing, validation and interpretability practices, and distinguishing evidence-based findings from future prospects. The overall framework of this review is illustrated in the following sections, organized by sensing modality and supplemented with architectural and methodological discussions.

## 2. Computer Vision and Image Recognition

Computer vision represents the most extensively adopted AI technical solution for tea quality assessment. Cameras capture visual information of tea samples; convolutional neural networks (CNNs) and derivative architectures process image data to realize automatic tea-grade classification, cultivar identification and defect screening. The emergence of YOLO-series object-detection frameworks and Transformer-based networks further enhances detection precision and computational efficiency for tea-related visual tasks. The general workflow of computer vision-based tea quality evaluation comprises image acquisition, preprocessing (background removal, color correction, image enhancement), data augmentation, feature extraction via deep neural networks, and quality prediction or classification, as shown in Table 2. The evolution from traditional machine learning with hand-crafted features (color histograms, texture descriptors, morphological parameters) to end-to-end deep learning has dramatically improved both accuracy and generalization capability across diverse tea varieties and imaging conditions.

### 2.1. CLIP-Powered Multimodal Grading Approaches

Long Tea CLIP is an expert-oriented multimodal artificial intelligence framework designed for fine-grained green tea grading across five sensory dimensions [34]. Built upon ResNet, XGBoost, multi-layer perceptron (MLP) and contrastive language-image pre-training (CLIP), this model was trained over datasets labeled by internationally acknowledged tea specialists. It assimilates visual signals originating from dry-tea morphology, tea-liquor color and post-infusion leaf appearances. In addition to these visual inputs, Long-Tea-CLIP also integrates chemical data characterizing aroma and taste, so that the five sensory dimensions (appearance, liquor color, aroma, taste and infused leaf) are jointly evaluated rather than inferred from vision alone. This distinguishes it from Tea MvMtNet (Section 7), a multi-view multi-task network that grades flavor factors exclusively from dry-tea, liquor and infused-leaf visual images. CLIP-supervised training lifted classification accuracy from 89.46% to 92.00%, constituting state-of-the-art performance for AI-facilitated tea-quality grading. Equipped with a bidirectional feature pyramid network augmented by attention modules, this model enables evaluation across all five sensory factors, including taste and aroma attributes [12,20]. The five-dimensional evaluation framework aligns with professional tea judging standards, potentially bridging the gap between AI-predicted scores and human expert assessments. Furthermore, the modular sub-architecture allows independent optimization of each sensory dimension before fusion, providing flexibility for deployment scenarios where certain imaging modalities may be unavailable.

### 2.2. Deep-Learning Implementations for Grading, Blending and Sample Matching

A refined grade classification model has been constructed for application in oolong tea and black tea grading, blending, and sample-matching workflows [35]. The model reached 95.05% classification accuracy for oolong tea samples and 99.13% for black tea materials, outperforming baseline networks EfficientNet, MobileNet and VGG16. For oolong tea blending tasks, the model yielded an average absolute error of 2.26%, surpassing manual sensory evaluation; black tea sample matching produced an average error magnitude of 3.34%. The practical significance of these results lies in the demonstrated feasibility of replacing or augmenting human sensory panels with AI-driven vision systems for industrial tea blending operations. The ability to perform sample matching with sub-5% error margins suggests that computer vision systems can maintain consistency in product standardization, a critical requirement for brand quality assurance in the tea industry. Notably, the model architecture employs multi-scale feature extraction to capture both fine-grained morphological details and broader textural patterns, addressing the heterogeneous visual characteristics of different tea categories [19,21].

### 2.3. YOLO-Series Object-Detection Architectures

YOLO (You Only Look Once) family detectors have been widely adapted for tea-bud identification and grading assignments [43,44]. TBC-YOLOv7 embedded Transformer modules into the original YOLOv7 backbone, integrating bidirectional feature pyramid structures and coordinate-attention components. It achieved 88.2% detection accuracy for one-bud-one-leaf (BOL) samples and 86.9% for one-bud-two-leaf (BTL) samples, with corresponding AP values equal to 90% and 85% [36].

An optimized YOLOv8 model replaced partial network segments with Swin Transformer (Hierarchical Vision Transformer using Shifted Windows) blocks to lower computational burdens while retaining detection performance [45]. An Efficient Multi-Scale Attention (EMA) module with cross-spatial learning capacities was embedded to strengthen feature extraction for fresh-tea-leaf grading scenarios.

More recently, an optimized YOLOv12 framework incorporated C3k2_EMA, A2C2f_DYT and RFAConv modules. For fine-grained fresh-tea-leaf detection and sorting, it attained 81.2% precision, 90.6% recall and mAP@0.5 exceeding 92% [38].

YOLO-PC was developed to simultaneously accomplish tea-bud pose estimation and grade discrimination. Dynamic snake convolution (DSConv) was adopted for morphological feature extraction; ELASPP-CSPC attention realized spatial-pooling operations. It obtained 91.5% accuracy for one-bud-one-leaf samples and over 93% accuracy targeting one-bud-two-leaf materials [39].

The progressive evolution from YOLOv7 through YOLOv8 to YOLOv12 illustrates a clear trajectory of integrating attention mechanisms and multi-scale feature fusion into real-time detection frameworks. A key trend is the replacement of standard convolution operations with deformable or attention-augmented variants (DSConv, C3k2_EMA, RFAConv) that better adapt to the morphological variability of tea buds and leaves. The YOLO-PC model’s joint pose estimation and grading capability is particularly noteworthy, as it enables robotic harvesting applications where spatial localization and quality assessment must be performed simultaneously. These advancements collectively demonstrate that the YOLO family, originally designed for general-purpose object detection, can be effectively specialized for the fine-grained morphological discrimination required in tea agriculture.

### 2.4. CNN-Driven Quality Identification

A research project targeting rapid green tea quality prediction constructed image datasets for three green tea morphological categories, namely flat-shaped (DFLJ), sparrow tongue-shaped (HSMF), and granular-shaped (PSRZ). ResNet18 and MobileNetV3 were validated for appearance-based grade discrimination [40]. A dual-branch fusion network was proposed to jointly execute tea-leaf disease diagnosis and age-dependent quality grading. Multi-class classifiers output four quality tiers (Q1–Q4), and composite multi-task loss functions supported end-to-end network training [41]. Fluorescence imaging coupled with CNNs was explored for tea-sample classification; under the experimental dataset, VGG16 delivered superior performance compared with ResNet-34 [31].

### 2.5. Knowledge Distillation for Fermentation-Quality Judgement

Knowledge distillation techniques have been introduced for the determination of black tea fermentation quality. Without increasing model complexity or slowing inference speed, the final model delivered 96.93% accuracy, 95.15% precision, 95.79% recall and 95.46% F1-score. Performance was improved by 2.01–3.72 percentage points compared with the original student baseline model [42]. Knowledge distillation offers a compelling strategy for deploying high-performance tea quality models on resource-constrained edge devices. By transferring knowledge from a large teacher network to a compact student model, this approach maintains competitive accuracy while reducing computational requirements, making it suitable for real-time fermentation monitoring on production lines where GPU resources may be limited; knowledge distillation has likewise been used to obtain drift-robust gas recognition in E-nose systems [44].

### 2.6. Transfer Learning and Data Augmentation Strategies

Transfer learning has emerged as a critical enabler for tea quality evaluation, addressing the persistent challenge of small dataset sizes typical in agricultural research. Pre-trained models on large-scale datasets (ImageNet, COCO) provide initialization points that significantly reduce training data requirements and accelerate convergence. Studies have demonstrated that models pre-trained on general food image corpora can be fine-tuned for tea-specific tasks with as few as 200–500 samples, achieving performance comparable to models trained from scratch with 2000+ samples [24]. Such sample-size claims, however, rest on single datasets and require independent confirmation before they can be generalized to other tea varieties or settings. Common transfer learning strategies include feature extraction (freezing backbone weights and training only classification heads), fine-tuning (updating all layers with differential learning rates), and domain-adversarial training to reduce distribution gaps between source and target domains. Data augmentation techniques play an equally important role in mitigating data scarcity. Beyond standard transformations (rotation, flipping, scaling, color jittering), advanced augmentation strategies including MixUp, CutMix, and generative adversarial network (GAN)-based synthesis have been explored for tea image datasets. Such region-mixing strategies are attractive for grading tasks because they combine regions drawn from different quality grades, which discourages the model from relying on global image statistics alone; for tea images, however, the benefit of CutMix has not yet been quantified against plain augmentation baselines. Additionally, generative-model-based augmentation approaches, exemplified by GAN-augmented agricultural image datasets for pest detection [46], offer a promising route to mitigate label scarcity in agricultural imaging; however, their application specifically to tea quality assessment remains limited.

### 2.7. Tea Defect Detection and Foreign Matter Screening

Beyond quality grading, computer vision techniques have been applied to tea defect detection and foreign matter contamination screening, which are critical for food safety compliance. Deep learning models have been trained to identify common tea defects, including insect damage, disease spots, mechanical damage, and discoloration. Semantic segmentation networks such as U-Net and DeepLab have been adapted for pixel-level defect localization, enabling precise identification of affected regions on individual tea leaves. For foreign matter detection, models have been developed to screen for contaminants such as metal fragments, hair, plastic particles, and non-tea plant materials. Real-time detection pipelines that couple high-speed imaging with lightweight CNN detectors have been developed for tea sorting, including foreign-object detection models for Pu-erh tea validated by five-fold cross-validation [18,22]; however, these studies report detection accuracy rather than throughput, and whether laboratory-level performance is sustained under real industrial-line conditions (including vibration, dust and variable illumination) remains to be confirmed. These defect detection systems often employ anomaly detection paradigms, where models trained on defect-free samples flag deviations as potential defects, reducing the need for comprehensive defect-labeled training datasets [47,48,49,50,51].

## 3. Electronic Nose (E-Nose) Technology

Electronic nose systems mimic human olfactory perception by deploying gas-sensor arrays to capture volatile aroma compounds released from tea specimens. Combined with machine-learning or deep-learning algorithms, E-nose devices support aroma-quality quantification, cultivar discrimination and geographical-origin tracing. The core components of an E-nose system include a sensor array exposed to tea volatile compounds, signal conditioning circuitry, data acquisition hardware, and pattern recognition software. Metal oxide semiconductor (MOS) sensors dominate tea quality assessment applications due to their low cost, high sensitivity, and diverse selectivity profiles. Emerging sensor technologies include conducting polymer sensors, quartz crystal microbalance (QCM) sensors, and surface acoustic wave (SAW) devices, each offering distinct sensitivity and selectivity characteristics for different volatile organic compound classes, as shown in Table 3. The coupling of E-nose systems with statistical and machine-learning pattern recognition for tea quality monitoring has been reviewed elsewhere [52], and combined SPME–E-nose analyses have linked specific aroma compounds to the quality of Bao-Chung tea [53]. Sensor array design involves selecting complementary sensors whose response profiles collectively span the chemical space of tea aroma compounds, typically including alcohols, aldehydes, ketones, esters, and terpenes.

A gas sensor array-based electronic nose system has been deployed in conjunction with deep learning workflows for tea quality assessment. Stacked sparse autoencoders constructed the neural-network backbone, which was trained via multi-layer optimization strategies [54]. This methodology mitigates well-known drawbacks associated with conventional sensory evaluation: subjectivity, evaluator fatigue, absence of real-time measuring capacity and susceptibility to measurement drift-induced errors. The stacked sparse autoencoder architecture offers advantages over traditional shallow classifiers by learning hierarchical representations of sensor response patterns. Each layer progressively extracts more abstract features, enabling the model to discriminate subtle aroma profile differences that may be indistinguishable through linear analysis methods. The multi-layer optimization strategy, combining layer-wise pre-training with global fine-tuning, addresses the vanishing gradient problem common in deep networks trained on relatively small E-nose datasets. Comparable chemometric workflows that couple E-nose responses with supervised classifiers such as PLS-DA and SVM remain a practical baseline for tea grading [28], and AI-assisted flavor blueprinting has recently been used to relate the aroma composition of tea infusions to sensory quality [55].

Even a single metal oxide semiconductor (MOS) gas sensor, when matched with appropriate deep learning architectures, has been shown to establish effective correlations between tea aroma chemistry and quality grades [56]. A multi-scale one-dimensional CNN integrated with Squeeze-Excitation units and temporal self-attention (MS-CNN-Attention) achieved macro-F1 = 0.811 under product-grouped cross-validation settings. It outperformed conventional feature-engineered pipelines (best macro-F1 = 0.624 obtained from MLP) by a margin of 0.187. The remarkable finding that a single MOS sensor can achieve effective quality grading when coupled with sophisticated deep learning architectures has significant implications for cost reduction in industrial E-nose deployment. The multi-scale CNN architecture captures both local transient dynamics and global temporal patterns in sensor response curves, while the Squeeze-Excitation mechanism adaptively recalibrates channel-wise feature importance. The 0.187 F1 improvement over traditional MLP approaches underscores the value of deep learning in extracting complex temporal dependencies from E-nose signals that traditional feature engineering fails to capture.

For geographical-origin classification tasks, E-nose hardware cooperating with machine learning algorithms discriminated oolong tea samples originating from four producing regions [57], namely Taiwan, Vietnam, China and Indonesia. Linear discriminant analysis (LDA), support vector machine (SVM), k-nearest neighbors (KNN) and other algorithms were compared, while grey relational analysis was used to conduct feature optimization [37]. Another comparative investigation evaluated multiple computational-intelligence algorithms for E-nose-based tea classification, offering references for embedded-hardware deployment selection [29]. Sensor drift remains a critical challenge for long-term E-nose deployment in tea quality assessment. MOS sensors experience baseline resistance shifts and sensitivity changes over time due to aging, environmental humidity fluctuations, and poisoning by irreversible adsorption. Current drift compensation strategies include component correction methods based on principal component analysis (PCA), domain adaptation techniques that align drifted sensor responses with reference distributions, and transfer learning approaches that adapt pre-trained models to drifted sensor data. Additionally, sensor array optimization through feature selection methods (sequential forward selection, genetic algorithms) helps identify the most drift-resistant sensor subset, improving long-term prediction stability [30,44,58].

## 4. Near-Infrared Spectroscopy (NIRS)

Near-infrared spectroscopy utilizes characteristic absorption bands originating from hydrogen-containing chemical groups (O-H, C-H, N-H) within the near-infrared spectral domain. It enables fast quantitative detection of moisture, tea polyphenols, caffeine, free amino acids and other major tea constituents. High detection sensitivity, hardware portability and compatibility with on-line monitoring render NIRS indispensable for real-time quality supervision throughout tea-manufacturing workflows [6,26]. Spectral preprocessing is a critical step in NIRS-based tea quality evaluation, as raw spectra contain systematic noise from light scattering, baseline shifts, and instrument variations. Common preprocessing methods include multiplicative scatter correction (MSC), standard normal variate (SNV), Savitzky–Golay (SG) smoothing and derivatives, and extended multiplicative signal correction [59,60], as shown in Table 4. These techniques individually or in combination remove physical scattering effects while preserving chemical absorption information, and spectral transformation strategies have likewise been used to optimize quantitative models, e.g., for chlorophyll-content prediction in tea leaves [61]. Wavelength selection algorithms, including competitive adaptive reweighted sampling (CARS), successive projections algorithm (SPA), and genetic algorithm-based selection, identify the most informative spectral bands for specific quality attributes, reducing model complexity and improving prediction robustness. The synergy of appropriate preprocessing and wavelength selection can improve prediction R-squared values by 0.05–0.15 compared to models using raw full-spectrum inputs [59,60,61]. The same combination of spectral preprocessing and chemometrics has been used to predict quality-related parameters in instant tea [62].

The FICSS-CNN-CSAM architecture has been reported to integrate wavelength selection modules and channel-spatial attention mechanisms within CNN blocks. It realized simultaneous multi-catechin-content prediction for black tea samples from NIRS spectral inputs [63]. This network consists of four functional sub-components, namely wavelength selector, feature-extraction backbone, attention module and prediction head. This work demonstrates the performance synergies achieved through the integration of attention-enhanced deep-learning architectures with spectroscopic datasets.

For white-tea adulteration detection, the GAF-2D-CNN pipeline converted raw near-infrared spectral signals into two-dimensional visual representations using Gramian angular field (GAF) encoding, followed by two-dimensional CNN processing for quantitative geographical-adulteration-ratio estimation [64]. This approach directly processes unprocessed spectral inputs and achieves high prediction accuracy for unseen random adulteration proportions [66]. The GAF encoding approach represents an innovative bridge between one-dimensional spectroscopic data and two-dimensional image processing architectures. By transforming spectral sequences into polar coordinate representations and subsequently into Gramian angular field matrices, this method enables the application of mature 2D CNN architectures to spectroscopic data, potentially capturing inter-wavelength correlation patterns that one-dimensional models may overlook.

NIRS combined with improved back-propagation artificial neural networks (J-BP-ANN) realized quality-grade discrimination for Yongchuan Xiuya tea. Calibration-set outcomes delivered Rc^2^ = 0.953 and RMSECV = 0.031; prediction-set outputs yielded Rp^2^ = 0.942 and RMSEP = 0.03 [67]. In a comparable single-instrument framework, NIRS was used both to identify the geographical origin of Lushan Yunwu tea and to predict its taste-related compounds [68]. Rapid detection hardware has been further developed by integrating visible/short-wave NIRS (400–1050 nm) with long-wave NIRS (1051–1650 nm). Multi-source data fusion and machine learning techniques supported tea-polyphenol-content prediction across multiple tea cultivars and leaf-position groups [65]. The integration of visible/short-wave and long-wave NIRS into a single detection system represents a significant hardware advancement, effectively broadening the spectral coverage to capture both electronic transition bands (visible region) and overtone vibration bands (NIR region) relevant to tea chemical composition. Portable and handheld NIRS instruments are increasingly explored for field-side tea quality assessment, with smartphone-coupled spectrometers and miniaturized near-infrared sensors showing promise for on-site quality screening at tea plantations and processing facilities. These portable systems, while offering slightly reduced accuracy compared to benchtop instruments, provide unprecedented accessibility for small-scale tea producers and rural cooperatives [69].

## 5. Hyperspectral Imaging (HSI)

Hyperspectral imaging simultaneously acquires spectral signatures and spatial distribution information, visualizing spatial variation of quality-relevant chemical properties across tea samples [47,51]. This technique exhibits distinct advantages for chemical-component quantification, fermentation-process tracking and quality-grade categorization. The data processing pipeline for HSI-based tea quality evaluation typically involves several stages: radiometric calibration converting raw sensor outputs to reflectance values, dark-current correction, spectral region-of-interest extraction, preprocessing (SNV, MSC, baseline correction), dimensionality reduction, and predictive modeling, as shown in Table 5. Principal component analysis (PCA) remains the most widely used dimensionality reduction technique; however, deep autoencoders and t-distributed stochastic neighbor embedding (t-SNE) have been explored for more nuanced feature compression. The high dimensionality of HSI data (often 100–300 spectral bands) necessitates effective feature selection to prevent the curse of dimensionality and reduce computational load for real-time applications. Spatial-spectral feature extraction architectures, which jointly leverage pixel-level spectral information and neighborhood spatial context, have demonstrated superior performance compared to pixel-wise spectral-only analysis, as they capture the spatial heterogeneity of quality-relevant chemical components across tea sample surfaces.

One integrated HSI-and-deep-learning framework predicted tea quality indices (tea polyphenols and catechins) for samples under diverse physical states (fresh tea leaves, dried finished tea, tea powder) harvested in spring, summer and autumn growing seasons [70,74]. Original reflectance spectra, harmonic features and wavelet-transform-derived descriptors were fused into multi-domain indices. XGBoost, Gaussian-process regression and CNN models were systematically benchmarked.

To quantitatively evaluate the extent of black tea fermentation, hyperspectral imaging (HSI) data were fused with human sensory reference information. Sample spectra were employed to reflect taste-related signals, color image outputs supplied visual information, and pH measurements together with porphyrin-based sensing array spectra characterized olfactory-relevant properties, thereby enabling multi-information fusion modeling [71]. A middle-level PCA compression strategy achieved 99% variable dimensionality reduction while retaining 95% prediction-set accuracy [63].

HSI was combined with PCA dimensionality reduction and ResNet-50 networks for tea quality classification. Transfer-learning and data-augmentation workflows yielded 86.15% accuracy; particle swarm optimization (PSO) lifted accuracy to 89.23%, and two-strategy PSO (TSPSO) further improved performance to 92.31% [72]. HSI inputs were coupled with PSO-optimized CNN-LSTM networks, achieving 96.78% test set accuracy for black tea fermentation quality assessment [26]. The progressive improvement from baseline transfer learning (86.15%) through single-strategy PSO (89.23%) to two-strategy PSO (92.31%) demonstrates the significant role of hyperparameter optimization in HSI-based deep-learning models. The TSPSO approach simultaneously optimizes network architecture parameters and spectral feature selection, addressing the coupled optimization problem inherent in HSI data analysis. The CNN-LSTM combination is particularly suited for HSI time-series data, where CNN extracts spatial-spectral features from individual hyperspectral cubes while LSTM captures temporal evolution patterns during fermentation processes.

Electronic tongue outputs and HSI measurements were fused through a Transformer-based graph network architecture for black tea geographical origin traceability [73]. PCA-filtered HSI feature maps fed into a spatial-enhanced Swin Transformer (SEST) module; a purpose-built graph-network module completed multi-source feature fusion, achieving 99.07% classification accuracy for origin traceability, the highest value reported among the multimodal-fusion studies surveyed here [73].

Earlier investigations targeting matcha sensory-quality assessment utilized hyperspectral microscope imaging (HMI) covering the 400–998 nm spectral interval. Competitive adaptive reweighted sampling (CARS), random-forest and partial-least-squares (PLS) algorithms cooperatively predicted appearance, liquor-color, aroma, taste and comprehensive sensory-quality scores [31].

## 6. Electronic Tongue (E-Tongue) Technology

Electronic-tongue devices deploy multi-sensor arrays that simulate human gustatory perception, detecting taste-active chemical compounds within tea infusion liquids. Coupled with machine learning pipelines, E-tongue hardware enables objective quantitative evaluation of tea taste quality [75]. E-tongue sensor technologies employed in tea quality assessment include voltammetric electrodes (platinum, gold, glassy carbon), potentiometric sensors (ion-selective electrodes), and electrochemical impedance spectroscopy-based sensors, as shown in Table 6. Polymer-modified electrodes, such as those incorporating polypyrrole, polyaniline, and polythiophene conducting polymers, offer tunable selectivity profiles for different taste-active compounds. The sensor array design principle mirrors that of electronic noses: by combining sensors with partially overlapping selectivity, the system generates a composite response pattern that serves as a chemical fingerprint of the tea infusion’s taste profile. Signal processing for E-tongue data typically involves baseline correction, normalization, feature extraction (statistical features, wavelet coefficients), and classification or regression modeling.

Electronic tongue-enabled tea quality evaluation has been systematically investigated. Nine polymer graphite composite electrodes (PGE) with varied acrylamide-aniline-pyrrole compounding ratios were fabricated for black tea sample characterization [33]. That research concentrated on sensor-hardware development and pattern-recognition workflows supported by machine learning methodologies.

Miniature near-infrared spectroscopic sensors and electronic-tongue detectors were integrated with multi-modal data-fusion strategies and chemometric tools to rapidly characterize black tea taste-quality attributes [75]. Grey wolf optimization (GWO), ant colony optimization (ACO), PSO and NSGA-II executed data-optimization operations; SVM, extreme learning machine and KNN algorithms constructed multi-grade prediction models.

A CNN-based automatic-feature-extraction workflow was designed for E-tongue-based tea classification. Sensor response signals were transformed into time-frequency maps using short-time Fourier transform (STFT), and CNN networks subsequently extracted discriminative features from 5100 sensor responses spanning five tea categories [74]. As noted in ref. [74], these 5100 sensor responses comprise repeated measurements and multiple concentration levels of a limited number of tea products rather than 5100 independent tea samples; this distinction should be borne in mind when judging model reliability. This approach demonstrates that signal-to-image transformation strategies can effectively expand the applicability of CNN architectures to non-image sensor data, a methodology also explored in NIRS and E-nose applications.

## 7. Multimodal Sensor Fusion

**Figure 2:**
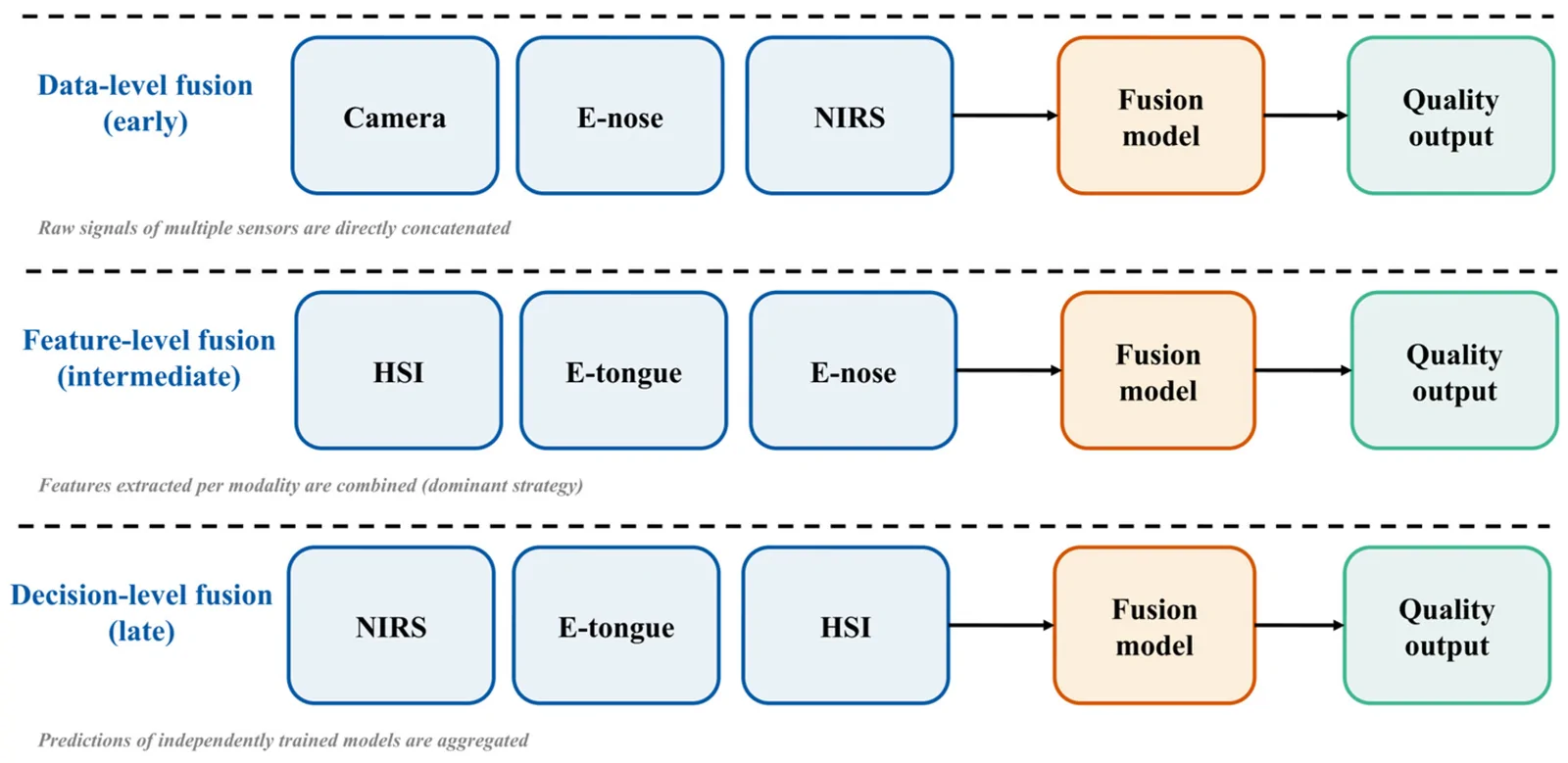
Schematic illustration of data-level (early), feature-level (intermediate) and decision-level (late) multimodal sensor fusion for tea quality evaluation.

Single-modality sensing technologies generally capture merely one dimension of tea quality characteristics, such as visual appearance, aroma information or taste signals. Multimodal sensor-fusion approaches aggregate multi-source sensor outputs to realize comprehensive quality profiling, representing one major frontier of contemporary tea-assessment research, as shown in Table 7. Sensor fusion strategies can be broadly categorized into three levels: data-level (early) fusion, feature-level (intermediate) fusion, and decision-level (late) fusion (Figure 2). Data-level fusion concatenates raw outputs from multiple sensors before any feature extraction, preserving maximum information but requiring aligned data acquisition and increased computational resources. Feature-level fusion combines extracted features from individual sensor modalities, offering a balance between information preservation and computational efficiency; this approach dominates current multimodal tea quality research. Decision-level fusion aggregates predictions from independently trained modality-specific models, offering modular flexibility but potentially losing cross-modal interaction information. Attention-based fusion mechanisms, which learn adaptive weights for different modalities based on their task relevance, have emerged as a promising alternative to fixed fusion strategies. Cross-modal attention modules enable one modality to guide feature selection in another, capturing complementary information that single-modality processing cannot exploit [77].

Tea MvMtNet is a multi-view multi-task deep neural network that processes visual signals collected from dry-tea morphology, brewed-liquor color and post-infusion leaf images. It estimates five sensory-flavor factors and completes tea-category classification [78,79]. This architecture adopts enhanced-attention bidirectional feature pyramid network modules, mitigating limitations of conventional algorithms which only process single-dimensional data and ignore the complex nature of tea-flavor formation. The multi-view design of Tea MvMtNet, encompassing dry tea, liquor, and infused leaf images, reflects the holistic nature of professional tea sensory evaluation where all three visual aspects contribute to quality judgment. The enhanced-attention bidirectional feature pyramid network enables the model to capture fine-grained visual cues at multiple scales, from overall color and shape to local texture and structural details. This architecture demonstrates that multi-view visual fusion alone, without incorporating chemical sensor data, can achieve comprehensive quality profiling approaching that of trained assessors in the reported study.

A dual-stream multimodal Transformer network fused PTR-ToF-MS volatile-metabolite measurements and targeted non-volatile-metabolite data for data-driven grading of Bai Mudan white tea samples [80,81]. The independent prediction set delivered 95.8% classification accuracy and a macro-average F1-score equal to 0.958. SHAP value analysis and attention-map visualization supplied interpretable insights regarding model decision-making logic. Coupling SHAP attribution with the multimodal Transformer made each grade assignment traceable to individual volatile and non-volatile metabolites rather than to the fused representation as a whole, which is the property that matters most for regulatory and industry acceptance; the attribution formalism itself is described in Section 10.2. The dual-stream Transformer architecture processes volatile and non-volatile metabolite data through separate encoder branches before fusion, allowing the model to learn modality-specific representations while the cross-attention mechanism captures inter-modal relationships [80].

For tencha (raw material for matcha) drying-process monitoring, a snapshot multispectral camera acquiring 25 spectral bands was applied. Attention-augmented deep-learning and dual-modal fusion workflows combined spectral-reflectance outputs and ten grayscale-texture descriptors inside attention-embedded SE-ResNet18 networks to implement chlorophyll content estimation [82].

**Table 7:** Summary of multimodal sensor fusion studies in tea quality evaluation.

Study	Modalities	Fusion Strategy	Architecture	Performance (as Reported)
Tea MvMtNet [78]	Dry tea + liquor + leaf	Feature-level	Multi-view DNN + BiFPN	Five flavor factors estimated jointly with tea-category classification
Dual-stream Transformer [80]	Volatiles + non-volatiles	Cross-attention	Transformer + SHAP	95.8% acc.; macro-F1 = 0.958
Tencha drying monitor [82]	Spectral + texture	Attention fusion	SE-ResNet18	Chlorophyll content estimated during drying
HSI + E-tongue TGNN [73]	HSI + E-tongue	Graph fusion	SEST + GNN	99.07% acc. (origin traceability)
HSI + sensory fusion [71]	HSI + color + pH + array	Multi-info fusion	PCA + ML	95% prediction-set acc.

## 8. Deep Learning Combined with Mass Spectrometry and Metabolomics

Apart from direct sensor-signal processing, deep learning techniques have been applied to chromatographic and mass-spectrometric datasets for tea-authenticity identification. A deep learning framework was constructed to convert liquid chromatography-mass spectrometry (LC-MS) metabolomic profiles into image-like representations. This model accomplishes robust Tieguanyin tea authentication under practical analytical lab conditions [52]. Investigators profiled 274 Tieguanyin specimens covering spring–autumn harvest seasons and light-scented/strong-scented processing variants. The proposed deep learning model reached 90.9% classification accuracy (95% CI: 80.4–96.0%), outperforming sPLS-DA (85.5%) and random forest (87.3%). Notably, under chromatographic-drift interference (a widespread source of analytical irreproducibility), the deep learning model retained 78.2% accuracy, whereas traditional chemometric approaches degraded to 69.1%, demonstrating superior anti-interference performance. The robustness of deep learning models against chromatographic drift has profound implications for real-world metabolomic tea authentication. Chromatographic drift encompasses retention time shifts, peak shape changes, and intensity variations that arise from column aging, mobile phase composition fluctuations, and temperature instability. Traditional chemometric methods typically require extensive data alignment and normalization to compensate for these effects, adding preprocessing complexity. This gap suggests that neural networks can learn drift-invariant features from metabolomic data, potentially reducing the need for meticulous chromatographic standardization. It should be noted that the reported drift resistance was assessed under simulated chromatographic drift (controlled perturbations of the analytical conditions) rather than under long-term, continuous drift encountered in multi-laboratory practice; performance under genuine long-term drift remains to be verified. This finding is particularly relevant for multi-laboratory studies and long-term monitoring programs where instrument drift is unavoidable. Furthermore, the conversion of LC-MS profiles into image-like representations enables the application of mature computer vision architectures to metabolomic data, facilitating knowledge transfer between domains.

## 9. Deep Learning Architecture Overview

**Figure 3:**
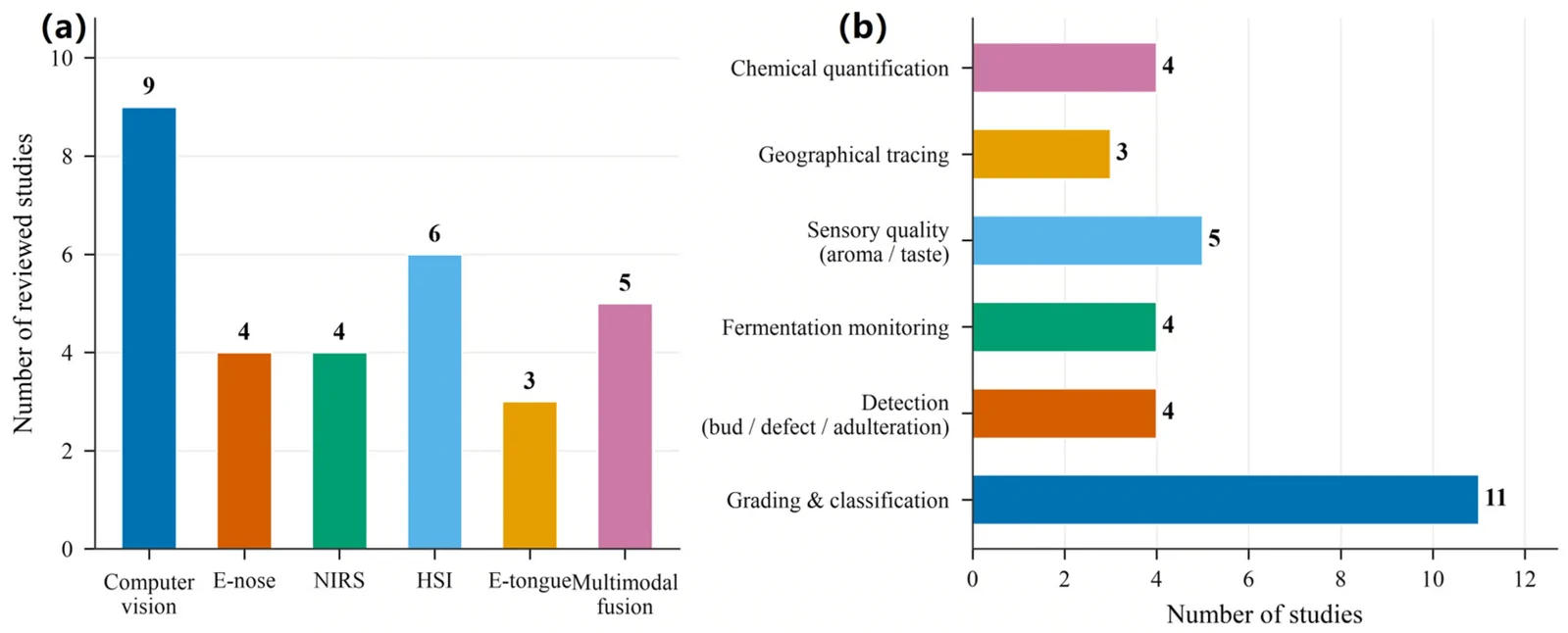
Overview of the reviewed literature. (**a**) Number of studies per sensing modality summarized from Table 2, Table 3, Table 4, Table 5, Table 6 and Table 7; (**b**) distribution of application tasks across the reviewed studies. Studies that address multiple modalities appear in more than one table; for example, refs. [71,73] are listed in both the HSI and the multimodal sensor-fusion sections. For the modality counts in (**a**), a study that appears in more than one table is counted once for each modality in which it is reported; consequently, the sum across modalities may exceed the number of unique studies.

The diverse tea quality evaluation applications reviewed in the preceding sections employ a range of deep learning architectures, each offering distinct advantages for different data modalities and task requirements. This section provides a systematic overview of the principal architectures and their roles in tea quality assessment. Figure 3 overviews the distribution of the reviewed studies across sensing modalities and application tasks.

It should be emphasized that the application tasks identified in Figure 3b are methodologically distinct. Origin identification, bud/leaf detection and chemical quantification are discriminative or regression tasks whose high reported performance is not, by itself, evidence of expert-level overall sensory assessment, which requires holistic grading across appearance, liquor color, aroma, taste and infused leaf under standardized protocols. Accordingly, high accuracy on a single task (e.g., origin tracing or defect detection) should not be equated with the ability to reproduce trained sensory-panel judgements of overall tea quality; the validation requirements and interpretability of such models differ accordingly across task types.

### 9.1. Convolutional Neural Networks (CNNs)

CNNs remain the foundational architecture for image-based tea quality evaluation. Classical architectures including VGG16, ResNet-18/34/50, MobileNetV3, and EfficientNet have been extensively validated for tea grading tasks. ResNet’s residual connections address the degradation problem in deep networks, enabling effective training of 50+ layer models that capture hierarchical visual features from low-level edges and textures to high-level shape and color patterns. MobileNetV3 and EfficientNet offer optimized depth-width-resolution trade-offs suitable for mobile and edge deployment. Recent advances integrate channel attention (SE blocks), spatial attention (CBAM), and multi-scale attention (EMA) modules into CNN backbones, adaptively emphasizing quality-relevant feature channels and spatial regions. These attention-enhanced CNNs consistently outperform their vanilla counterparts by 2–5% accuracy across tea grading, defect detection, and cultivar identification tasks [83].

### 9.2. Transformer Architectures

Transformer-based models, originally developed for natural language processing, have been increasingly adapted for tea quality evaluation. Vision Transformers (ViT) and Swin Transformers process image data through self-attention mechanisms that capture long-range spatial dependencies, complementing CNNs’ local feature extraction. In tea quality assessment, Swin Transformer modules embedded within YOLO architectures enhance multi-scale feature representation for bud detection and grading tasks. Dual-stream Transformer networks process volatile and non-volatile metabolite data through parallel encoder branches, with cross-attention mechanisms capturing inter-modal interactions. The self-attention mechanism’s ability to model global dependencies is particularly valuable for E-nose and E-tongue time-series data, where long-range temporal correlations in sensor response patterns carry critical quality information. However, Transformers typically require larger training datasets than CNNs, making transfer learning and data augmentation essential strategies in tea quality applications where data availability is limited [84].

### 9.3. Graph Neural Networks (GNNs)

GNNs model relational structures within tea quality data through message passing along graph edges. In the context of tea quality evaluation, GNNs have been employed for multimodal sensor fusion, where different sensor modalities (HSI, E-tongue, E-nose) are represented as nodes and their relationships as edges in a graph structure. The Transformer-based graph network for black tea origin traceability exemplifies this approach, where PCA-filtered HSI features and E-tongue measurements are processed through graph convolution layers that aggregate information across sensor nodes. GNNs offer advantages over traditional concatenation-based fusion by explicitly modeling inter-modality relationships and enabling information propagation between sensor modalities. This relational modeling capacity is particularly relevant for tea quality assessment, where the interactions between visual appearance, aroma, and taste attributes collectively define overall quality [84].

### 9.4. CLIP and Foundation Models

The Long Tea CLIP framework represents the application of foundation model paradigms to tea quality evaluation. CLIP (Contrastive Language-Image Pre-training) learns shared embedding spaces for visual and textual descriptions, enabling zero-shot or few-shot classification through natural language prompts. In the tea domain, this approach allows models to leverage expert-level sensory descriptions as training signals, bridging the semantic gap between visual appearance and quality attributes. The contrastive learning objective trains the model to align visual features of tea samples with their corresponding textual quality descriptions, creating a unified representation space where both modalities can inform quality predictions. Foundation models pre-trained on large-scale multimodal datasets provide strong initialization points, and fine-tuning on tea-specific data has been reported to achieve high performance with relatively small labeled datasets, as exemplified by the Long Tea CLIP framework for green tea grading [35]. This paradigm is expected to gain further traction as pre-trained models become more accessible and tea-specific multimodal datasets expand. Because the reviewed studies differ in task, dataset and metric, their reported performance is compiled study by study in Table 2, Table 3, Table 4, Table 5, Table 6 and Table 7 instead of being aggregated into route-level means: each entry now gives the metric, the number of independent samples and the validation scheme where the source reports them, so that comparison is restricted to studies that address the same task with the same metric.

## 10. Data Processing and Model Interpretability

Beyond architectural innovations, effective data processing pipelines and model interpretability methods are essential for developing trustworthy and deployable AI-based tea quality evaluation systems. This section reviews key methodological developments in these areas.

### 10.1. Data Preprocessing and Augmentation

Tea quality evaluation data from diverse sensor modalities require modality-specific preprocessing strategies. For computer vision, image preprocessing includes background subtraction (using thresholding or segmentation networks), color constancy correction (to compensate for lighting variations), and geometric normalization. For spectroscopic data (NIRS, HSI), preprocessing involves scatter correction (MSC, SNV), derivative computation (Savitzky–Golay), and wavelength selection (CARS, SPA) [50,51]. For E-nose and E-tongue signals, baseline correction, response normalization, and feature extraction from temporal response curves are standard practices [74]. Data augmentation is critical for addressing the small dataset problem in tea quality research. Image augmentation strategies include geometric transformations, color space perturbations, and advanced techniques like MixUp, CutMix, and GAN-based synthesis [36,38,45]. For spectral data, augmentation through noise injection, spectral shifting, and generative modeling helps expand limited datasets. Cross-domain augmentation, where models pre-trained on one tea variety are adapted to another through domain adaptation techniques, provides another avenue for data-efficient model development.

### 10.2. Model Interpretability Methods

As deep learning models increasingly inform tea quality decisions, understanding their decision-making processes becomes critical for regulatory compliance and industry acceptance. SHAP (SHapley Additive exPlanations) values provide a unified measure of feature importance, quantifying the contribution of each input feature to individual predictions; the formalism was developed for tabular models and has been applied, for instance, to the classification and nutritional-content prediction of different meat cuts [58]. In tea grading, SHAP attribution has been used to identify the volatile and non-volatile metabolites that drive the assigned grade, providing a basis for domain-expert validation [79]. Attention visualization, naturally available in Transformer and attention-augmented CNN architectures (e.g., the Long Tea CLIP framework and YOLO-based detectors), reveals which spatial regions, spectral bands, or temporal segments the model focuses on for quality predictions [22,26]. Grad-CAM (Gradient-weighted Class Activation Mapping) generates heatmaps overlaying input images, highlighting discriminative regions for tea grade classification. LIME (Local Interpretable Model-agnostic Explanations) provides local surrogate models that approximate complex neural network decisions about specific predictions. These interpretability methods collectively transform black-box deep-learning models into transparent decision support systems, fostering trust among tea industry stakeholders and facilitating model debugging and improvement.

### 10.3. Validation Strategies

Robust validation is essential for ensuring that reported model performance generalizes to real-world deployment scenarios. Beyond standard hold-out validation, several advanced validation strategies have been employed in tea quality evaluation research. Product-grouped cross-validation, where samples from the same product batch are kept together in either training or testing sets, provides a more realistic estimate of generalization performance than random cross-validation. Independent external testing using samples from different harvest seasons, geographic origins, or processing facilities offers the most stringent validation. Leave-one-variety-out cross-validation assesses generalization across tea varieties, while temporal validation, where models trained on historical data are tested on subsequently collected samples, evaluates temporal stability. Among the studies reviewed here, product-grouped cross-validation, in which samples from the same product batch are kept together in either the training or the test set, was explicitly used in the single-MOS-sensor aroma-grading study (macro-F1 = 0.811); this design prevents the over-optimistic inflation that can arise when repeated measurements from the same product appear in both training and test partitions. Independent prediction sets were employed in the multimodal white tea grading study [78] and in the LC-MS-based Tieguanyin authentication study, whereas random data splits remain the most common practice in the remaining studies. Where studies rely on repeated measurements (e.g., the 5100 e-tongue responses in ref. [74]), samples from the same product could appear in both training and test sets if the split is performed at the measurement level rather than the product level; this should be avoided or explicitly reported. More generally, claims of performance superiority are only meaningful when the compared models are evaluated under identical data splits, sample sets and metrics. The adoption of standardized benchmark datasets and evaluation protocols would greatly facilitate cross-study performance comparison and accelerate progress in the field.

## 11. Existing Challenges and Future Research Perspectives

Despite substantial research progress, several critical bottlenecks impede the translation of AI-based tea quality assessment technologies from laboratory settings to real-world industrial deployment. These include the limited size and lack of standardization of existing datasets, which hinders cross-study performance comparison, alongside the restricted interpretability of deep learning models due to their black-box nature. Additional challenges involve sensor drift and stability issues in electronic nose and tongue systems that compromise long-term reliability, as well as poor cross-scenario generalization arising from variations in tea categories, geographic origins, and seasonal factors. Furthermore, the high costs of hyperspectral imaging hardware, multi-sensor platforms, and GPU computing resources constrain adoption among small- and medium-sized enterprises, while the gap between laboratory-scale experiments and actual production line conditions remains largely unresolved. It should also be noted that some reviewed studies report exceptionally high accuracies (>95%). Because many tea datasets are small and single source, such figures may partly reflect favorable data splits and limited test diversity rather than intrinsic model superiority, and raise concerns about overfitting; they should therefore be interpreted cautiously until confirmed by independent external validation on samples from different harvest seasons, origins or facilities (Section 10.3). In addition, the sensor signals exploited by the reviewed systems correspond to specific chemical classes (e.g., NIRS/HSI bands for polyphenols and catechins, e-nose responses for volatile aroma compounds, and e-tongue signals for taste-active compounds); for models trained on human sensory scores, predictions can only be as reliable as the reference scores themselves. Panel size, assessor training and inter-/intra-assessor agreement should therefore be reported and considered before describing such models as ‘objective’ or ‘expert-level’, whereas models trained on instrumental ground truth (e.g., quantified chemical markers) provide a firmer basis for objectivity claims. Beyond these well-recognized challenges, several additional issues warrant attention. First, the lack of standardized benchmark datasets and evaluation protocols makes it difficult to fairly compare different approaches; many studies report results on proprietary datasets that are not publicly available, creating reproducibility concerns. Second, most of the current research focuses on single tea varieties or narrow quality grade ranges, with limited investigation into cross-variety generalization. Third, real-time deployment requirements demand not only model accuracy but also computational efficiency, yet many proposed architectures involve complex attention mechanisms and multi-branch structures that may not achieve the throughput speeds required for industrial production lines. Fourth, sensor calibration and maintenance protocols for long-term deployment remain underexplored, particularly for E-nose and E-tongue systems operating in tea factory environments with fluctuating temperature, humidity, and dust conditions. Finally, regulatory acceptance of AI-based quality assessment tools requires standardized validation frameworks and certification procedures that do not yet exist for tea quality evaluation, necessitating collaborative efforts between researchers, industry stakeholders, and regulatory bodies.

To address these constraints, future research should prioritize the construction of large-scale, standardized benchmark datasets that encompass diverse tea varieties, origins, and harvest seasons, alongside the development of interpretable multimodal modeling workflows incorporating attention mechanisms and explainable AI tools. Lightweight network designs suitable for edge computing and real-time inspection should be pursued through knowledge distillation and neural architecture search, while innovative architectures such as Transformers, CLIP, and graph neural networks warrant further exploration. Interoperable multi-sensor hardware platforms with modular and plug-and-play functionality are also needed, as are engineering-oriented investigations to bridge the gap between laboratory research and production-line deployment. Additionally, emerging research directions include the integration of foundation models pre-trained on large-scale food and agricultural datasets, enabling transfer learning to tea-specific tasks with minimal labeled data. Federated learning approaches could facilitate collaborative model training across multiple tea research institutions without sharing proprietary data, addressing data privacy and intellectual property concerns [77]. It should be emphasized that these emerging technologies, in particular foundation models and federated learning, are currently prospective rather than experimentally validated in tea quality assessment; the reviewed evidence for them is largely conceptual or drawn from other domains, and substantial validation on tea-specific datasets is required before they can be regarded as deployable. The development of tea-specific data augmentation pipelines, including physically based spectral simulation and GAN-generated synthetic tea images, could help overcome dataset scarcity. Edge-cloud collaborative computing architectures, where lightweight models perform initial screening on-device and complex analysis is offloaded to cloud infrastructure, offer a practical pathway for industrial deployment. Finally, the establishment of tea quality assessment benchmarks and competitions, like ImageNet for general computer vision, would stimulate community engagement and drive rapid progress in the field. In parallel, strict validation pipelines, including product-grouped cross-validation and fully independent external testing, must be widely adopted to ensure that reported performance metrics generalize reliably to practical applications.

## 12. Conclusions

This review examines artificial intelligence-enabled tea quality evaluation across six technological streams: computer vision, electronic nose, near-infrared spectroscopy, hyperspectral imaging, electronic tongue, and multimodal sensor fusion. The literature indicates that deep learning architectures, including CNNs, YOLO detectors, Transformers, CLIP, and graph neural networks, when coupled with diversified sensing hardware, have achieved classification accuracies between 85% and 99% across tasks such as grade sorting, aroma identification, fermentation monitoring and origin tracing, alongside strong quantitative prediction of chemical constituents. The research trajectory has progressed from isolated single-technology proof-of-concept experiments toward multimodal integration, explainable algorithm development, and deployment-oriented investigations. The systematic analysis of deep learning architectures reveals that attention mechanisms, multi-scale feature extraction, and cross-modal fusion strategies are key enablers of the high-performance levels reported across different tea quality tasks. The emerging application of foundation models, particularly CLIP-based frameworks, represents a promising direction towards holistic quality profiling; however, expert-level performance has so far been demonstrated only on specific datasets. Model interpretability methods, including SHAP analysis, attention visualization, and Grad-CAM, are increasingly integrated into proposed frameworks, addressing the critical transparency requirements for industrial and regulatory acceptance.

Nevertheless, persistent challenges, including dataset standardization deficiencies, insufficient model interpretability, sensor stability limitations, weak cross-domain generalization, and high implementation costs, must be resolved before AI-based analytical tools can become standardized quality evaluation instruments. With continued expansion of dataset scale, innovation in deep learning architectures, and improvements in sensor hardware, intelligent perception-driven detection systems may progressively become mainstream tea quality assessment tools over the coming years, provided that dataset standardization, external validation, sensor stability and hardware-cost challenges are resolved, thereby gradually complementing rather than replacing manual sensory panel evaluation. The future trajectory of this field will likely be shaped by several converging trends: the maturation of foundation models enabling data-efficient learning, the proliferation of low-cost portable sensors expanding accessibility, the advancement of edge computing enabling real-time on-device inference, and the establishment of standardized benchmarks facilitating rigorous performance comparison. As these technological and methodological advancements converge, AI-driven tea quality evaluation systems are poised to transition from laboratory research to widespread industrial adoption, fundamentally transforming how tea quality is assessed, certified, and traded in the global market.

## Figures and Tables

**Table 1:** Comparison of traditional and AI-based tea quality evaluation methods.

Method	Objectivity	Throughput	Destructiveness	Cost	Scalability	Real-Time
Sensory evaluation	Low	Low	Non-destructive	Medium	Poor	No
HPLC/GC-MS	High	Low	Destructive	High	Limited	No
Computer vision + DL	High	High	Non-destructive	Low	Good	Yes
E-nose + DL	High	Medium	Non-destructive	Medium	Good	Yes
NIRS + DL	High	High	Non-destructive	Medium	Good	Yes
HSI + DL	High	Medium	Non-destructive	High	Moderate	Limited
Multimodal fusion	High	Medium	Non-destructive	High	Moderate	Limited

**Table 2:** Summary of computer vision-based tea quality evaluation studies.

Study	Architecture	Application	Tea Type	Accuracy (as Reported)
Long Tea CLIP [34]	ResNet + CLIP + XGBoost	5-dimension grading	Green tea	92.00%
Grading/blending model [35]	Custom CNN	Grading & blending	Oolong/Black	95.05%/99.13%
TBC-YOLOv7 [36]	YOLOv7 + Transformer	Bud detection	Fresh leaves	88.2%/86.9%
Improved YOLOv8 [37]	YOLOv8 + Swin + EMA	Leaf grading	Fresh leaves	92%+ mAP
Enhanced YOLOv12 [38]	YOLOv12 + C3k2_EMA	Fine-grained sorting	Fresh leaves	92%+ mAP
YOLO-PC [39]	YOLOv7 + DSConv	Pose & grading	Fresh leaves	91.5%/93%+
ResNet18/MobileNetV3 [40]	CNN	Grade prediction	Green tea	85%+
Dual-branch fusion [41]	Dual-branch CNN	Disease & grading	Tea leaves	Q1–Q4
KD fermentation model [42]	Distilled CNN	Fermentation quality	Black tea	96.93%

**Table 3:** Summary of E-nose-based tea quality evaluation studies.

Study	Sensor Type	Algorithm	Application	Performance (as Reported)
Stacked SAE E-nose [54]	Gas sensor array	Stacked sparse autoencoder	Quality assessment	Quality discrimination improved relative to conventional sensory evaluation
Single MOS sensor [25]	Single MOS	MS-CNN-Attention	Aroma-quality grading	macro-F1 = 0.811 (product-grouped CV)
Oolong origin classification [37]	E-nose array	LDA/SVM/KNN + GRA	Geographical tracing	Discrimination of four producing regions
Algorithm comparison [29]	E-nose array	Multiple AI methods	Tea classification	Comparative evaluation; metrics vary by method

**Table 4:** Summary of NIRS-based tea quality evaluation studies.

Study	Spectral Range	Algorithm	Application	Performance (as Reported)
FICSS-CNN-CSAM [63]	NIR	CNN + attention	Catechin prediction	R^2^ = 0.92–0.97 and RMSE = 0.018–0.32 for EC, ECG, EGC and EGCG (four catechins); *n* = 105 independent samples (70 calibration/35 prediction); 10-fold CV with an independent prediction set
GAF-2D-CNN [64]	NIR	GAF + 2D-CNN	Adulteration detection	R^2^ = 0.9754 and RMSE = 0.0349 on untrained random adulteration ratios; external verification on held-out adulteration levels
J-BP-ANN [38]	NIR	Improved BP-ANN	Grade discrimination	Rp^2^ = 0.942
Vis/SW + LW NIRS [65]	400–1650 nm	ML fusion	Polyphenol prediction	Polyphenol prediction across cultivars and leaf positions

**Table 5:** Summary of HSI-based tea quality evaluation studies.

Study	Spectral Range	Algorithm	Application	Performance (as Reported)
Multi-domain HSI + DL [70]	HSI	XGBoost/GPR/CNN	Quality indices	R^2^ = 0.81 (tea polyphenols) and 0.76 (catechins) for autumn tea powder; 10-fold CV across season × physical-state sample sets
HSI + sensory fusion [71]	HSI + color + pH	PCA + fusion	Fermentation degree	95% prediction-set accuracy
HSI + ResNet-50 + PSO [72]	HSI	ResNet-50 + TSPSO	Quality classification	92.31%
HSI + CNN-LSTM [26]	HSI	PSO-CNN-LSTM	Fermentation quality	96.78%
HSI + E-tongue + TGNN [73]	HSI + ET	Transformer + GNN	Origin traceability	99.07%
HMI matcha assessment [31]	400–998 nm	CARS + RF + PLS	Sensory quality	Multi-attribute sensory-score prediction

**Table 6:** Summary of E-tongue-based tea quality evaluation studies.

Study	Sensor Type	Algorithm	Application	Performance (as Reported)
PGE electrode array [33]	Polymer graphite	ML methods	Black tea charact.	Sensor characterization; metric not reported (doctoral thesis)
Mini NIR + ET fusion [75]	NIR + E-tongue	GWO/ACO/PSO + SVM	Taste quality	93.56% correct classification rate (ACO–SVM, seven grades); n = 700 independent samples (467 calibration/233 prediction); Kennard–Stone split with prediction-set validation; NIR (3 scans) and E-tongue (4 runs) replicates averaged per sample
STFT-CNN classifier [76]	E-tongue	STFT + CNN	Tea classification	5-category classification; 5100 responses are repeated measurements, not independent samples

## Data Availability

No new data were created or analyzed in this study. Data sharing is not applicable to this article.

## Abbreviations

The following abbreviations are used in this manuscript:

CNN	convolutional neural network
YOLO	you only look once
CLIP	contrastive language-image pre-training
HPLC	high-performance liquid chromatography
GC-MS	gas chromatography-mass spectrometry
MLP	multi-layer perceptron
BTL	one-bud-two-leaf
EMA	efficient multi-scale attention
DFLJ	flat-shaped
HSMF	sparrow tongue-shaped
PSRZ	granular-shaped
LDA	linear discriminant analysis
SVM	support vector machine
KNN	k-nearest neighbors
GAF	Gramian angular field
HSI	hyperspectral imaging
SEST	spatial-enhanced Swin Transformer
LC-MS	liquid chromatography-mass spectrometry
BOL	one-bud-one-leaf
NIRS	near-infrared spectroscopy
MOS	metal oxide semiconductor
PCA	principal component analysis
PSO	particle swarm optimization
GNN	graph neural network
GAN	generative adversarial network
SHAP	SHapley Additive exPlanations
STFT	short-time Fourier transform
mAP	mean average precision
RMSE	root mean square error
MSC	multiplicative scatter correction
SNV	standard normal variate
PTR-ToF-MS	proton transfer reaction time-of-flight mass spectrometry
Grad-CAM	gradient-weighted class activation mapping
LIME	local interpretable model-agnostic explanations
